# Orientational bistability and field-controlled switching of a superparamagnetic dimer

**DOI:** 10.1039/d6sm00349d

**Published:** 2026-08-14

**Authors:** James R. N. Tett, Finlay Johnston, Brennan Sprinkle, Alice L. Thorneywork

**Affiliations:** a Physical and Theoretical Chemistry Laboratory South Parks Road Oxford OX1 3QZ UK alice.thorneywork@chem.ox.ac.uk; b Department of Applied Mathematics and Statistics, Colorado School of Mines Golden CO USA

## Abstract

We study the orientational dynamics of superparamagnetic colloidal dimers that carry both an induced magnetic moment, proportional to the applied field, and an effective permanent moment. In a static, uniform magnetic field, dimers that are permanently fixed together hop between two preferred in-plane angles, developing a bimodal steady-state orientation distribution. When the same field is periodically reversed, we observe a sharp, field-controlled change in the dynamical response from small hopping events with Δ*θ* ≪ π to full Δ*θ* ≈ π rotations on each field flip. We show that both the static bistability and the switching bifurcation can be rationalised by a magnetic response in the dimer that consists of both a strong induced and weak body-fixed component. This leads to a complex orientational energy landscape, with coupled roll-yaw rotations of the dimer responsible for the bistable dynamics. By combining the misorientation between dimer axis and field, bifurcation field strength and short-time orientational variance, we determine the magnitude and orientation of the net permanent dipole, thereby characterising details of the internal magnetic structure of the particles *via* microscopy.

## Abstract 2

We study the orientational dynamics of superparamagnetic colloidal dimers containing both induced and effective permanent moments, revealing bistable dynamics and field-controlled switching between distinct rotational responses.

## Graphical abstract

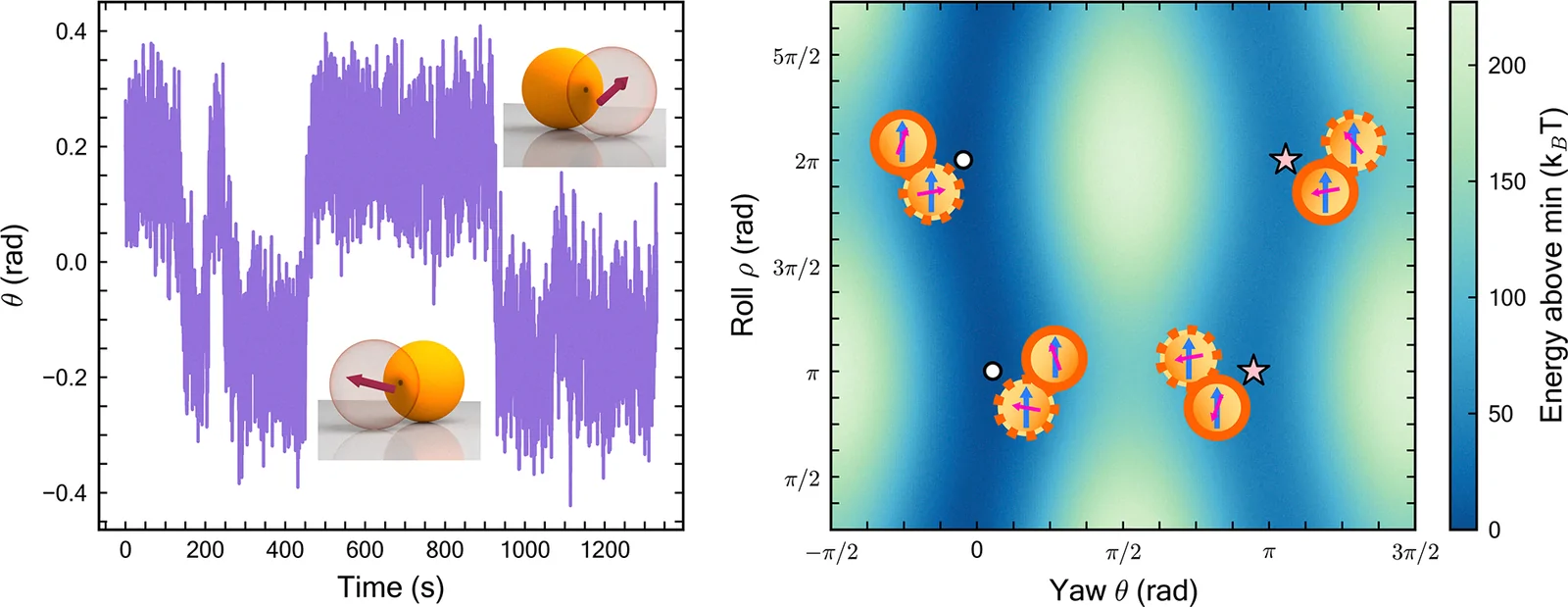

Suspensions of superparamagnetic microparticles (SPMPs) find varied applications in nano- and biotechnology, serving as microactuators,^1–3^ biosensors,^4^ and transducers in single-molecule magnetic tweezing.^5,6^ More fundamentally, they represent a versatile soft matter model system that has field-tunable, anisotropic interactions.^7–9^ This range of applications has motivated extensive effort to understand how magnetic field protocols control the structure and dynamics of particle assemblies, from effective pair interactions^8^ and field-driven chaining^10^ to collective organisation^11^ and transport in confined geometries.^12^ In this context, magnetic dimers, *i.e.* an assembly of just two particles, are the simplest non-trivial structural component.^13^

In many colloidal and biophysical settings, SPMPs are modelled as hard spheres with a purely induced dipole moment that is proportional to, and instantaneously aligned with, the applied field.^14–16^ In static, uniform fields, this induced-dipole picture is generally sufficient to rationalise forces and effective interaction potentials.^15,17–19^ Yet in many applications, the goal is to apply a magnetic torque to the bead; a process that is not possible in the above picture as it requires misalignment between the bead's net magnetic moment and the local field. As such, for rotating fields, the response is more subtle, involving magnetic anisotropy and/or internal degrees of freedom. Despite routine use of torque, for example, in magnetic tweezers, a full understanding of the bead-specific torque response remains elusive.^6,18,20^

Complexity around this question ultimately originates from the internal structure of SPMPs: a dilute dispersion of iron-oxide nanoparticles (NPs) embedded in a polymer matrix (Fig. 1).^21^ In an idealised scenario, on application of an external field the embedded nanoparticles respond independently. The orientation of each magnetic moment is then governed by the competition between Zeeman coupling to the applied field and magnetocrystalline anisotropy, relative to the thermal energy *k*_B_*T*. In the superparamagnetic limit, where thermal fluctuations overcome the relevant anisotropy barriers on the experimental timescale, the ensemble response gives a net induced moment *μ*_ind_ ∝ *B* that is, on average, aligned with **B** and vanishes when the field is removed.

**Fig. 1:**
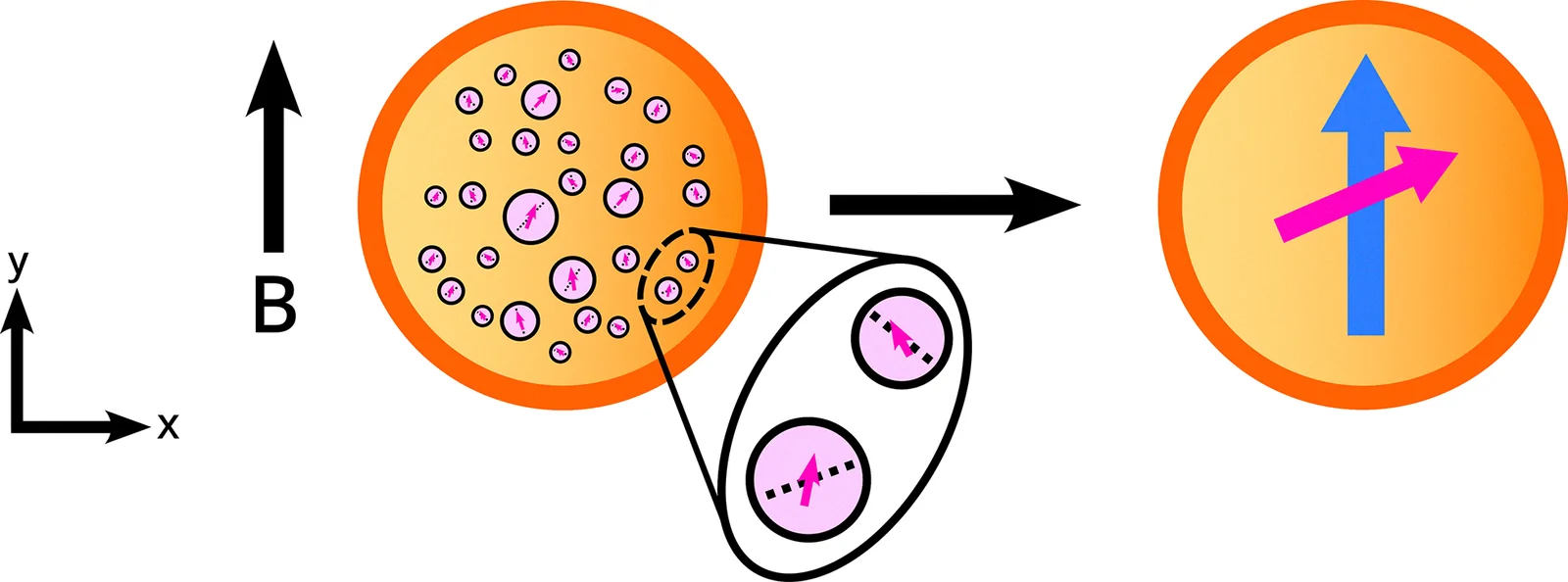
Illustration of the internal structure and magnetic response of a superparamagnetic microparticle in an external field. Nanoparticles with net magnetic moments (lilac) show a random spatial distribution. Associated with these is a magnetic easy axis (dashed line). In an external field, the collective response has two contributions: (i) a dominant induced moment, *μ*_ind_ (blue), aligned with the applied field **B**, and (ii) a weaker body-fixed magnetic moment, represented as an effective permanent moment, *μ*_p_ (pink), fixed in the particle frame at a constant angle to the particle director.

If a subset of nanoparticle moments have large anisotropy barriers compared with *k*_B_*T* on the experimental timescale, however, their moments may become blocked. These barriers can arise from the magnetocrystalline anisotropy of individual NPs and are enhanced when neighbouring NPs are close enough to interact and no longer behave as independent superspins.^22,23^ In particular, interacting clusters can possess large effective anisotropy barriers and preferred magnetic axes.^23,24^ At the coarse-grained level, this can give rise to the appearance of a weak, body-fixed moment superimposed on the dominant induced response; in effect, an apparent permanent moment (Fig. 1). Experiments on commercial beads report both permanent contributions that can dominate low-frequency rotation^25,26^ and an effective easy-axis anisotropy that becomes apparent at higher field strengths,^18^ with details of the permanent contribution varying across measurements.^18,20,26^ Many studies focus on high fields, however, where the induced moment dwarfs any residual contribution. In contrast, the weak-field regime, where even small body-fixed contributions can compete with the induced response to influence interaction potentials and dynamics, is less well explored.

Here, we show that the orientational dynamics of superparamagnetic dimers in weak external fields reveals details of internal magnetic structure. We first explore behaviour in a static field for three different particle assemblies and confirm that the orientational dynamics of these different assemblies is consistent with coexisting induced and permanent moments in the weak-field regime. The field dependence of the effective interaction provides a scaling argument that distinguishes an effective permanent moment from anisotropic susceptibility effects. For two superparamagnetic particles rigidly fixed together we find that the dimer hops between two preferred orientations; a phenomenon that can be explained by considering three-dimensional rotation of the net permanent moment. This establishes a complex two-dimensional potential landscape for dimer dynamics linked to coupled roll-yaw rotations of the dimer. Next, we consider how a dimer explores this complex landscape in response to flipping of the field direction. With increasing field magnitude, we find a sharp transition between two qualitatively different relaxation dynamics and again link this behaviour to details of the underlying landscape. For both static and flipping fields, we find quantitative agreement between experiment and theory, with these combined measurements allowing for the effective permanent dipole of individual dimers to be quantified from microscopy alone.

## 1 Experimental methods

Our colloidal dimers were built from carboxylate-functionalised superparamagnetic beads of diameter *σ* ≃ 3 µm (Dynabeads M-270). These particles are reported to have an iron-oxide weight fraction of order 14%.^27^ Suspensions were prepared at low volume fraction in deionised water and loaded into sealed glass sample cells of height *H* ≈ 200 µm. Rigid magnetic–magnetic dimers are found at low concentration in all samples and arise from irreversible surface interactions (*e.g.* short range van der Waals attraction) between two beads, giving a centre–centre separation ℓ ≈ *σ*.^28^ To increase the proportion of such permanently bound dimers and aggregates, samples were prepared in a high concentration of divalent ions, centrifuged and then re-dispersed in deionised water before imaging (see Section S1). This treatment screens electrostatic repulsion between functionalised particle surfaces, promoting a short-range attraction that leads to irreversible aggregation. Hybrid magnetic–non-magnetic dimers were prepared by mixing streptavidin-functionalised magnetic beads (Dynabeads M-280) with 2.8 µm carboxylate-functionalised melamine-formaldehyde spheres (MF-COOH-AR811, MicroParticles GmbH) *via* the same protocol.

When loaded into the sample cell, beads sedimented to the lower surface and the resulting out-of-plane fluctuations were small compared to *σ* (gravitational height *h*_g_ ≈ 65 nm ≪ *σ*),^27^ so that the translational and orientational dynamics were quasi-two-dimensional. With respect to rotations, this leads to a strong gravitational suppression of pitch fluctuations of the dimer axis out of the sedimentation plane. A uniform, in-plane magnetic field **B**(*t*) was applied using a pair of coaxial Helmholtz coils driven by a programmable current source. We used both static fields, **B** = *B***ŷ**, and square-wave protocols in which the field alternated between ±*B***ŷ** with period *T*. The field amplitude was calibrated as a function of coil current *via* a Hall probe and the field reversal time (∼2 s) was small compared to the characteristic rotational relaxation time of the dimers.

Samples were imaged in bright-field using an inverted microscope equipped with a high-numerical-aperture objective and a Thorlabs CMOS CS135MU camera (Fig. 2a shows an example image). Time series were acquired at 25 fps for up to 3 hours, sufficient to resolve intra-well Brownian fluctuations and the slower switching and flipping dynamics in static fields and field reversal. Particle coordinates were extracted using standard tracking methods.^29^ From tracked bead positions, we obtained the dimer centre-of-mass and in-plane orientation *θ*(*t*), defined as the yaw angle between the dimer axis and applied field direction, **ŷ** (Fig. 2b).

**Fig. 2:**
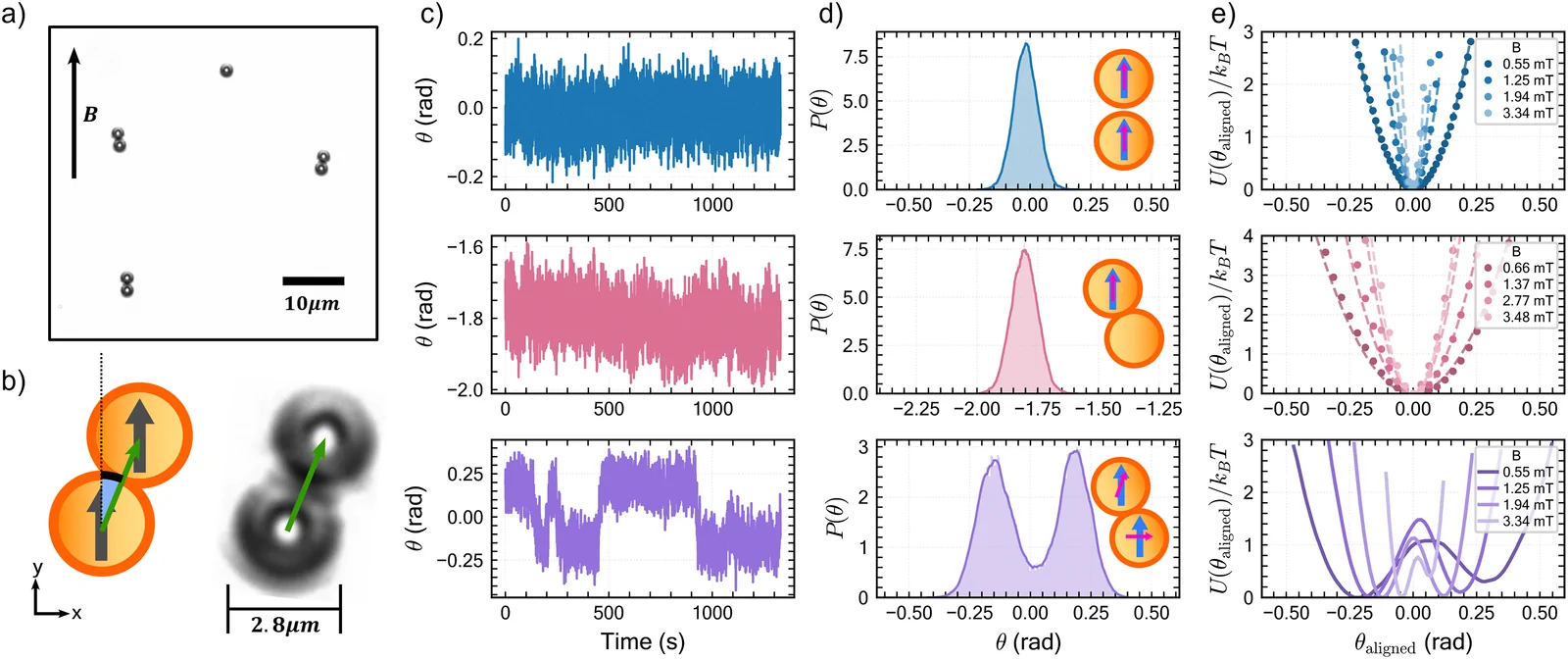
(a) Section of a typical experimental image showing magnetic dimers in an external field. The dimers shown are reversibly bonded such that removing the external field removes the attractive interaction. (b) Schematic of a magnetic–magnetic dimer showing the definition of the dimer axis and in-plane (yaw) angle with respect to the field. (c) Typical in-plane angle trajectories, *θ*(*t*) and (d) corresponding probability distributions *P*(*θ*) for: (top) two superparamagnetic particles interacting only *via* the field; (middle) a permanently bonded magnetic–non-magnetic dimer; (bottom) a permanently bonded magnetic–magnetic dimer. Data is taken at comparable field strengths (*B* = 1.25 mT for magnetic–magnetic dimers and *B* = 1.37 mT for magnetic–non-magnetic dimers). (e) Effective interaction potentials, *U*(*θ*) = −*k*_B_*T* ln *P*(*θ*) + const. for (top) two superparamagnetic particles interacting only *via* the field; (middle) a permanently bonded magnetic–non-magnetic dimer; (bottom) a permanently bonded magnetic–magnetic dimer. Potentials in panel (e) are centred to align their preferred (average) orientations, allowing comparison of the potential shape and curvature across field strengths. (top) and (middle): Points show experimental data and dashed lines are fits to the dipole–dipole and dipole–field interaction potentials, eqn (1) and (3), respectively. (bottom) Solid lines show the experimental effective interaction potential.

To quantify the rigidity of the bonded dimer assemblies, we tracked both constituent beads and computed the instantaneous bond length ℓ(*t*). The distribution of ℓ was narrowly peaked, with standard deviation comparable to the positional tracking uncertainty (∼15 nm, <1% of a bead diameter), and showed no systematic dependence on field strength, indicating that bond-length fluctuations are minimised and that the particles are rigidly bound together. Over the same trajectories, dimer centres exhibited diffusive in-plane motion with 〈Δ*r*^2^(*t*)〉 ≈ 4*Dt* (Fig. S2), consistent with free Brownian translation in the sedimentation plane. The mean squared angular displacements (MSADs, Fig. S3) were also proportional to *t* at short times, consistent with angular diffusion within a potential well.

## 2 Results and discussion

### 2.1 Dimer dynamics in static fields

We first consider the orientational dynamics of individual assemblies in a static uniform field **B** = *B***ŷ**, where **B** is the magnetic flux density. From the tracked bead positions, we obtain the in-plane yaw angle *θ*(*t*), defined relative to the applied field direction. Fig. 2(c) shows representative yaw trajectories for three cases: (i) a pair of magnetic beads held together by induced dipole–dipole interactions (reversible bonding), (ii) a permanently linked magnetic–non-magnetic dimer, and (iii) a permanently linked magnetic–magnetic dimer. In cases (i) and (ii), the yaw angle *θ*(*t*) fluctuates around a single mean orientation; for (ii), this mean is generally offset from the applied-field direction. In contrast, rigid magnetic–magnetic dimers exhibit intermittent switching between two orientations.

From long trajectories at fixed *B*, we construct the corresponding yaw distributions; these are shown in Fig. 2(d) as *P*(*θ*). For pairs bound only by magnetic interactions (top) and magnetic–non-magnetic dimers (middle), the distributions are unimodal. By contrast, for rigid magnetic–magnetic dimers, the distribution is bimodal across the measured field range. Rather than showing a single orientation aligned with the field, as expected for purely field-aligned induced dipoles, the distribution develops two preferred orientations separated by a lower-probability region near the mean angle. Whilst hydrodynamic mobilities differ between dimer types, this affects relaxation and switching timescales, rather than the equilibrium orientational landscape. As such, these differences in angular distribution imply a different energy landscape.

To visualise the effective angular landscape, we construct *U*(*θ*) = −*k*_B_*T* ln(*P*(*θ*)) + const. from the measured angular distribution and plot *U*(*θ*) for each dimer type at several field strengths (Fig. 2e). For the dimer interacting only *via* the magnetic field (top), experimental data (circles) are in good agreement with the expected form of the landscape arising from an induced dipole–dipole interaction between two moments aligned with **B** (lines) given by:

1 *U*_ind_(*θ*) = −*μ*_ind,1_·**T**_12_(*θ*)·*μ*_ind,2_,

where the bead-centre separation vector is **r̂**(*θ*) = sin(*θ*)**x̂** + cos(*θ*)**ŷ** such that the dipolar coupling tensor can be written explicitly as:2
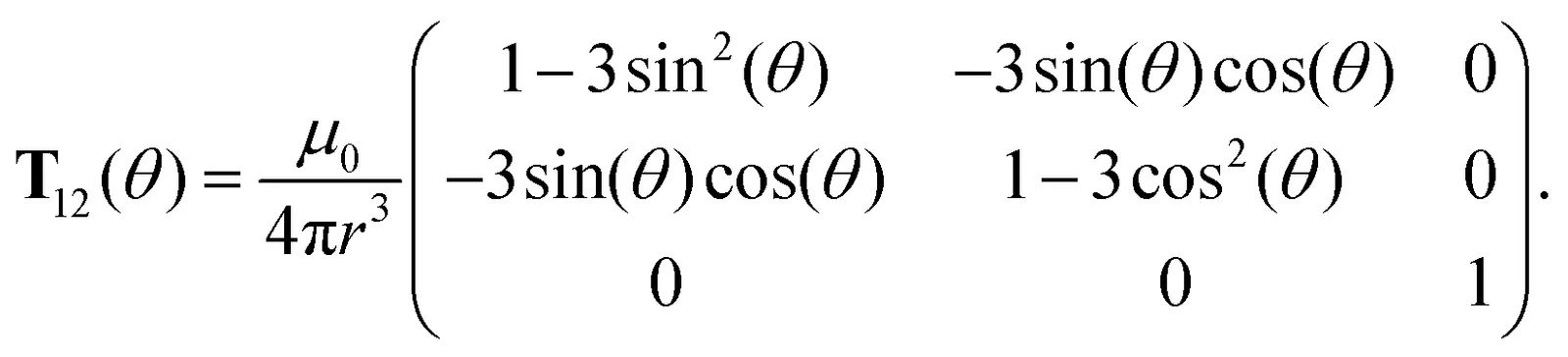


The induced moments are linked to the external field *via* a magnetic susceptibility *χ*, such that *μ*_ind,*i*_ = *χB***ŷ** in the isotropic, linear-response regime. Agreement between the experimental data and theory in Fig. 2e (top) to leading order indicates that an induced dipole-only picture is sufficient to describe the behaviour of magnetic–magnetic dimers without a rigid bond.

For the magnetic–non-magnetic dimer, a purely induced moment that is strictly collinear with **B** would produce no torque in a uniform field, and so there should not be a preferred angle for the dimer. The observed preferred yaw angle therefore requires a body-fixed contribution, consistent with the particle carrying a weak residual or permanent moment on experimental timescales.

As an initial model, we quantify this moment by fitting the magnetic–non-magnetic landscapes (Fig. 2(e), middle) to a 1D, effective permanent-dipole form:

3 *U*_hyb_(*θ*) = −*μ*_p_*B* cos(*θ* − *α*),

where *θ* is the observed yaw angle of the dimer relative to the applied field, *μ*_p_ is an effective body-fixed moment (*i.e.* a coarse-grained odd-in-*B* contribution fixed in the particle frame) and *α* is the orientation of the effective permanent moment with respect to the dimer axis (see Fig. S8 for the moment geometry). This one-dimensional, effective description captures both the unimodal shape of the measured distributions in Fig. 2(d) and (e) and is consistent with the fact that (i) dimers are observed to rotate by 180° upon field inversion (Fig. S6) and (ii) have an interaction strength proportional to *B*, rather than *B*^2^ (Fig. S7). These features distinguish the odd-in-*B* contribution from induced magnetisation, which, even in the presence of anisotropic susceptibility, should remain even-in-*B* and, in the linear-response regime, should scale proportional to *B*^2^. From the fits to data for the magnetic–non-magnetic dimer in Fig. 2(e), we obtain a linear scaling of interaction strength with field and *μ*_p_ ∼ (0.5–5) × 10^−16^ A m^2^ for individual particles. This is consistent with previously reported permanent-moment magnitudes for commercial superparamagnetic beads.^26,30^

For rigid magnetic–magnetic dimers, the bimodal *P*(*θ*) again indicates that an additional ingredient is at play beyond the induced interaction. While a natural first hypothesis is that the two peaks reflect two independent body-fixed moments, one in each bead, any combination of two permanent moments in the plane of the sample can be expressed in terms of a single effective permanent moment; the vector sum of the two bead-level moments. The interaction between the effective moment and applied field yields only a single minimum (Section S8.1). Rationalising the bimodal distribution, therefore, requires a fuller consideration of the orientation of this effective permanent moment. Although the dimer itself is confined to diffuse in a 2D plane, the effective permanent moment could have any three-dimensional orientation within the particle. As the dimer diffuses, it is therefore possible for the permanent contribution to move out of plane due to a roll about the dimer axis (see Fig. 3(a), where we denote the roll angle as *ρ*). This roll is not directly observable in our images due to the particle symmetry. For most orientations of the permanent moment, a change in *ρ* changes the projection of the permanent moment in the plane of the sample, and thus the preferred in-plane, or yaw, angle.

**Fig. 3:**
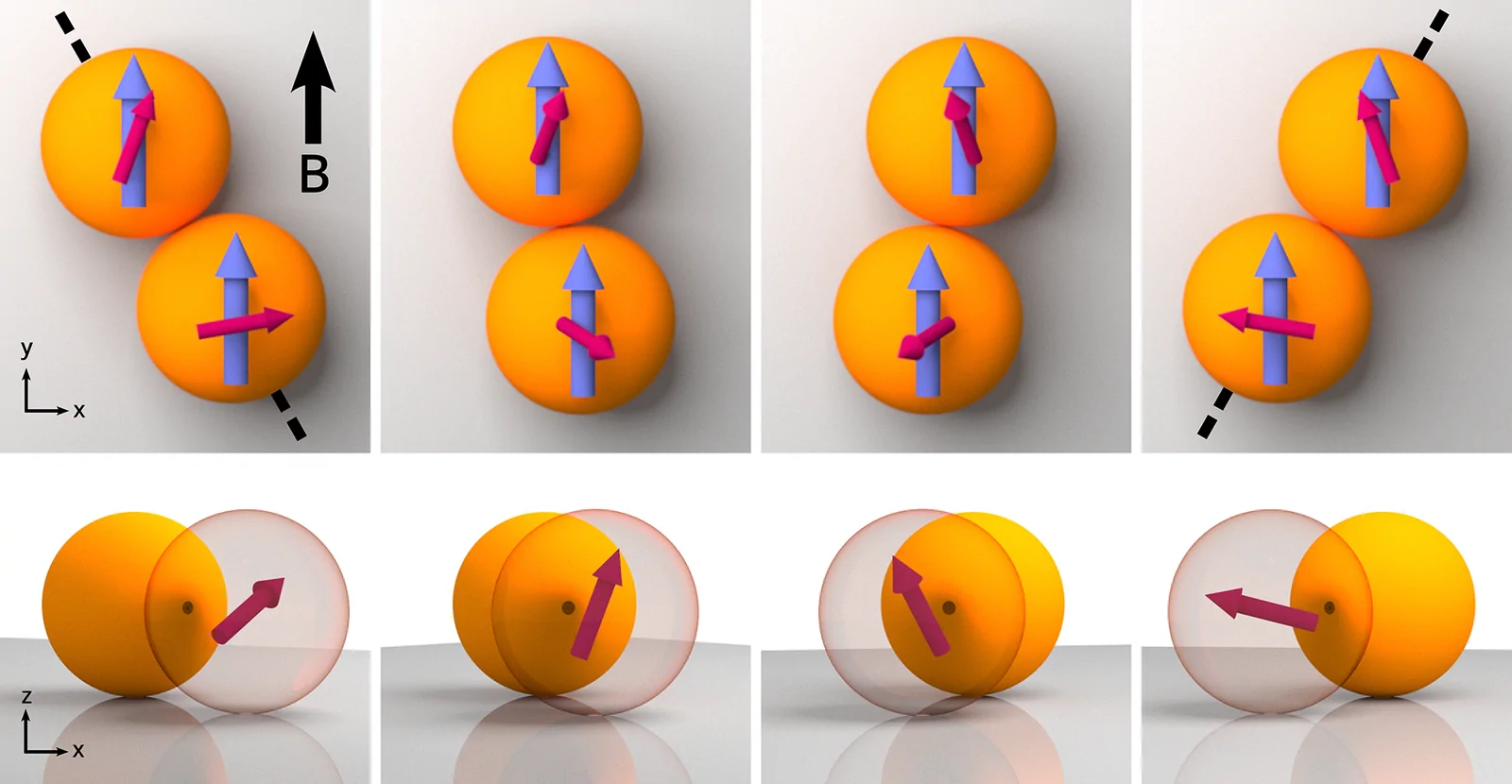
Schematic sequence (left to right) showing the continuous reorientation of a rigid dimer through coupled roll and yaw rotations when viewed from above (upper row) and in the sedimentation plane (lower row). Roll states are shown *via* the permanent moment orientation. Only one permanent moment is shown for clarity (lower row).

#### 2.1.1 Roll-yaw landscapes for rigid magnetic dimers

To understand the bimodal *P*(*θ*) distributions, we hypothesise that the measured distributions arise as a marginalised projection of a coupled (*θ*,*ρ*) landscape, and so explore the full roll-yaw energy landscape *U*(*θ*,*ρ*) of the dimer in detail. To construct the landscape, we modify eqn (1) to include the interaction between the permanent moment and external-field term as:

4 *U*(*θ*,*ρ*) = −*μ*_p,net_(*θ*,*ρ*)·**B** − *μ*_ind,1_·**T**_12_(*θ*)·*μ*_ind,2_,

where *μ*_p,net_ is the magnitude of the dimer's net permanent moment. This is the vector sum of the two, bead-level permanent moments, and can have arbitrary magnitude and orientation with respect to the dimer axis. The induced and permanent terms are even and odd in *B* respectively and mutual induction and permanent-induced cross terms produce only small corrections in our geometry (see Section S5).

Eqn (4) predicts a family of energy landscapes. The features of these landscapes depend on the relative values of induced and permanent magnetic interactions, as set by the external field and the magnitude and orientation of the permanent moments within each bead. Notably, these energy landscapes are applicable to any sedimented, rigidly-bound dimer with induced and permanent bead-level moments. Fig. 4(a) shows a typical example of such a landscape with experimentally reasonable values *B* = 1 mT and [|*μ*_p,net_|, arg(*μ*_p,net_)] = [2.6 × 10^−16^ A m^2^, 70°]. Within one 2π × 2π unit cell of the angular coordinates, this example landscape contains two global minima (marked as circles) related by the glide symmetry *U*(*θ*,*ρ*) = *U*(−*θ*,*ρ* + π) and two local minima (marked as stars), also related by a glide symmetry.

**Fig. 4:**
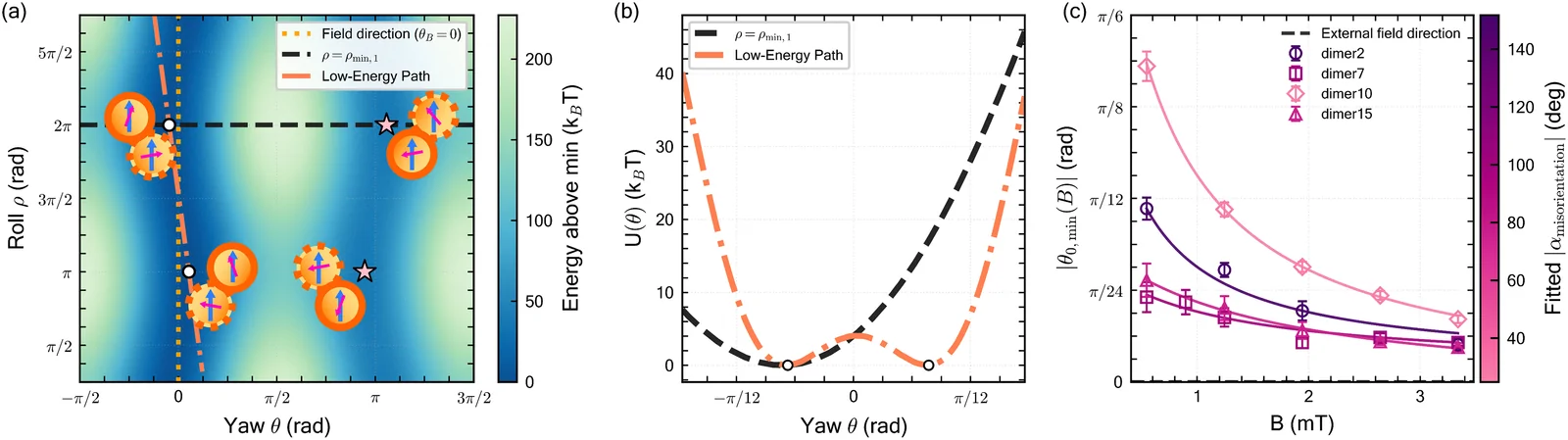
(a) Example 2D roll-yaw energy landscape *U*(*θ*,*ρ*;*B*) for a rigid dimer in a static field applied along *θ* = 0 rad. Circles mark the positions of global minima (permanent moments broadly aligned with the external field). Stars show the position of local minima (permanent moments broadly misaligned with the external field). Representative dimer configurations are shown for minima. (b) 1D cuts through the landscape in (a) corresponding to an in-plane, yaw only rotation (black dashed line) and a coupled roll-yaw path (orange dashed line). (c) Magnitude of the yaw-minimum displacement, |*θ*_min_(*B*)|, relative to the applied-field direction as a function of applied field strength, *B*, for multiple different dimers. Points show experimental data and lines show fits according to eqn (6), plotted as the corresponding magnitude of the predicted yaw-minimum position.

Global minima correspond to configurations where the permanent moment is broadly aligned with the applied field, whereas higher-energy local minima correspond to configurations in which the permanent moment is comparatively misaligned with the field. The energy barrier separating the global-minimum branch from the higher-energy local-minimum branch exceeds 40*k*_B_*T* for the parameters shown, so transitions between these branches are effectively inaccessible for a fixed field orientation on experimental timescales. Such large absolute energy scales are expected for the full magnetic landscape: even a permanent moment of 10^−16^ A m^2^ in a 1 mT field corresponds to a Zeeman energy of *μ*_p_*B*/*k*_B_*T* ≈ 24, while the induced dipole–dipole interaction scales as (*χ*^2^*B*^2^)/*r*^3^ and can reach hundreds to thousands of *k*_B_*T* over the full orientational space. These large energies should therefore be distinguished from the much smaller barriers inferred from the experimentally sampled, effective yaw potential in Fig. 2(e). The latter describe the low-energy projected pathway explored thermally by the dimer, rather than the full range of thermally inaccessible roll-yaw configurations.

We therefore consider thermally accessible pathways between minima within a 2π × 2π unit cell of angular coordinates, which would be sampled in our experimental, effective potentials. Starting from one of the global minima within the unit cell, marked by circles in Fig. 4(a), a horizontal section of the landscape, taken by varying only the yaw angle *θ* at fixed roll angle *ρ* = *ρ*_0_, contains only one thermally accessible minimum (black dashed line in Fig. 4(b)). By contrast, a diagonal path (orange), along which both *θ* and *ρ* vary, connects the two symmetry-related global minima through a thermally accessible barrier of order 4*k*_B_*T*. This supports our physical interpretation of the dimer hopping as arising from coupled roll-yaw motion.

Intuitively, the symmetry relation between these global minima can be understood as the effect of physical confinement on the orientational motion of the dimer. In an unconstrained three-dimensional geometry, a dimer carrying both induced and permanent moments could explore a continuous set of orientations in which the dimer axis maintains the same cone half-angle with respect to the applied field. In our sedimented geometry, this orientational manifold is intersected by the sample plane, and pitch rotations of the dimer axis are strongly suppressed by gravity and wall confinement. This results in only two accessible, symmetry-related yaw minima, with movement between these only *via* coupled roll and yaw rotations.

#### 2.1.2 Misorientation angle

Next, we seek to explain quantitatively the characteristic behaviour of the bimodal angular landscape as a function of field strength in Fig. 2(e), focussing on how the displacement of the yaw-minimum from the applied field varies with field strength. From the experimental *P*(*θ*) distributions, we extract the magnitude of the yaw-minimum position |*θ*_min_(*B*)|, relative to the applied-field direction. These values are plotted as a function of field strength for four different dimers in Fig. 4(c). At high fields, |*θ*_min_(*B*)| tends to a 1/*B* scaling, while at lower fields, the measured minima deviate from this asymptotic form. The magnitude of the measured misorientation is dimer-specific, reflecting differences in the magnitude and orientation of the net permanent moment between dimers.

To compare our experimental data quantitatively to the roll-yaw landscapes, we use a reduced 1D yaw potential obtained by conditioning on a particular roll state, *ρ* = *ρ*_min_, corresponding to the branch of the full roll-yaw landscape that contains the observed yaw minimum:

5 *U*_eff_(*θ*,*ρ* = *ρ*_min_;*B*) = −*κB*^2^ cos(2*θ*) − *μ*_p,net_*B* cos(*θ* − *α*)

where *κ* is the induced dipole–dipole interaction stiffness and *α* is the effective misorientation of the net permanent moment relative to the dimer axis on this roll branch (see Section S8). As the dimer rolls about its axis, the effective permanent moment traces a cone of half-angle *α*. In the full roll-yaw landscape, the laboratory-frame projection of the permanent moment changes with *ρ* but the misorientation of the permanent moment to the dimer axis *α* is a constant. By conditioning on *ρ*_min_ (field-independent in our geometry) in eqn (5), we consider one global minimum as a function of field. Applying the glide symmetry generates the global minimum on the second roll branch, recovering the paired minima observed experimentally (Fig. 2e). We note that eqn (5) has the same mathematical structure as the Stoner–Wohlfarth energy,^31^ with the induced dipole–dipole interaction providing the effective two-fold anisotropy and the permanent moment providing the Zeeman-like tilt.

On differentiation, eqn (5) yields the desired expression for the positions of potential minima as a function of applied field as

6 2*κB* sin(2*θ*_min_) + *μ*_p,net_ sin(*θ*_min_ − *α*) = 0

In principle, eqn (6) contains three unknowns: the induced dipole–dipole interaction stiffness, *κ*, the in-plane misorientation of the net permanent moment relative to the dimer *α* and the net permanent moment, *μ*_p,net_. The stiffness, *κ*, can, however, be determined from short-time intra-well fluctuations 〈Δ*θ*^2^〉 (see Section S7). As such, fitting eqn (6) to individual dimer data determines two dimer-specific parameters *μ*_p,net_ and *α*, on a dimer-by-dimer basis, example fits are shown in Fig. 4c. Measurement of intra-well fluctuations for rigid magnetic–magnetic dimers, yields a magnetic susceptibility of (1–10) × 10^−12^ A m^2^ T^−1^, consistent with literature measurements.^27^ Calculated magnetic moments are on the order (0.5–3.0) × 10^−16^ A m^2^, likewise in line with dimer literature measurements,^26,32^ with misorientation angle |*α*| ∼ (0.3–2.2) rad. It should be noted that small misorientation angles (*α*) are difficult to determine with this method, as dimer axis measurements will be masked by noise.

### 2.2 Dimer dynamics in flipping fields

Having characterised how the presence of a permanent moment influences the dynamics of rigid dimers in a static field, we now consider dimer dynamics in a flipping field. In particular, we apply a square-wave protocol, in which the field alternates between ±*B***ŷ** with a period much longer than the rotational relaxation time for the dimer. For purely induced dipole interactions, flipping the field by π leads to a corresponding switch in the direction of the induced dipole within the bead, and thus should not lead to rotation. In contrast, for the permanent moment, flipping the field direction will generate a more or less favourable situation, depending on whether the permanent moment is initially net aligned or misaligned with the field.

Fig. 5(a) and (b) shows the possible relaxation responses observed for rigid dimers in response to a field flip. At lower |*B*| (pink), the dimer rotates by ∼180° on each field reversal, separate from the small-angle hopping observed in a static field. At higher |*B*| (blue), the dimer executes small-angle hops between two static-field wells at ±*θ*_0_(*B*). In both cases, we observe both clockwise and anticlockwise rotations, in line with the sequence of minima in the energy landscape. Example experimental trajectories, showing the angle of the dimer as a function of time, can be seen in Fig. 5(b). At intermediate fields (purple), we find a mixed scenario with trajectories exhibiting both small-angle hops and full rotation by 180°. Remarkably, the transition in behaviour between full rotations and small hops occurs over a very small range of field strengths.

**Fig. 5:**
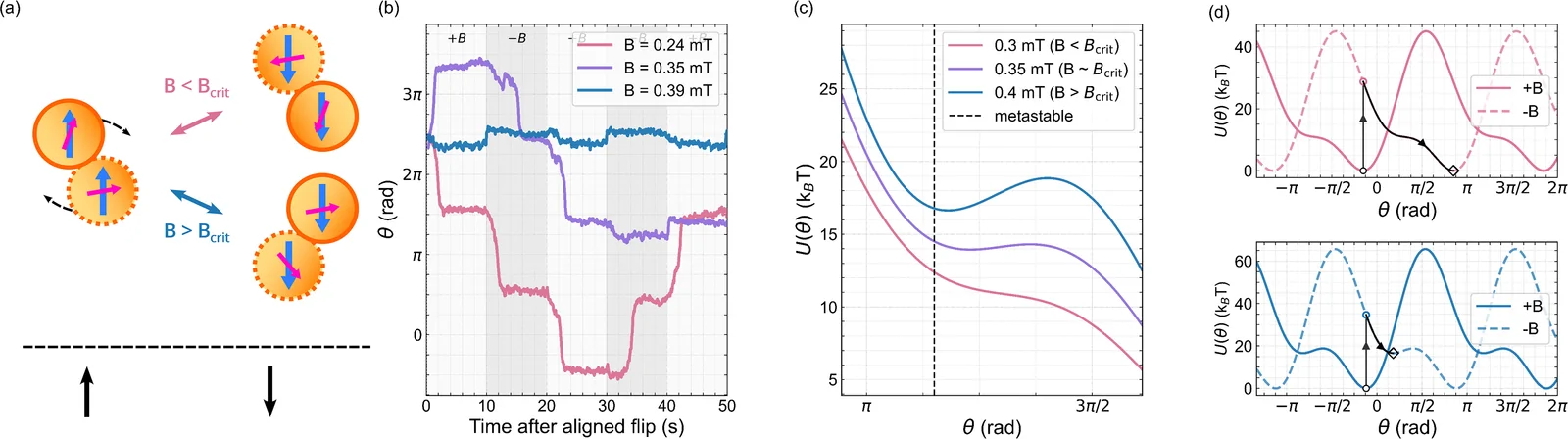
(a) Schematic showing the two dimer responses to field flips. Below the bifurcation field, the dimer undergoes full π reorientations (full flips) whilst above the bifurcation field, the dimer undergoes small reorientations (small hops). (b) Experimental angular trajectories for the same rigid dimer above (blue), around (purple) and below (pink) the critical field, *B*_crit_. (c) 1D cut-throughs of an example roll-yaw landscape, *U*(*θ*,*ρ* = *ρ*_min_), for increasing field magnitude. Note the development of a metastable minimum in the blue potential as the field passes through the bifurcation. (d) Relaxation under field reversal: 1D effective yaw potentials *U*(*θ*,*ρ* = *ρ*_min_) before and after field reversal, +*B* → −*B*, for field magnitudes below (top) and above (bottom) the bifurcation field. Below *B*_crit_, reversal drives relaxation by a large ∼π reorientation, whereas above *B*_crit_ the appearance of a metastable minimum leads to a small-angle hop.

These different trajectories can also be understood in terms of the roll-yaw landscape *U*(*θ*,*ρ*;*B*) (eqn (4)). Upon field reversal *B* → −*B*, the instantaneous configuration of the dimer is mapped to a high-energy region of *U*(*θ*,*ρ*;−*B*) (Fig. 5(d), (top)), and the dimer relaxes toward a new stable minimum. For low field strengths, *i.e.*, relatively weak induced moment interactions, the permanent moment (odd-in-*B* term) dominates, leading to large ∼π reorientation. As *B* increases, the strength of the induced moment grows, and the post-flip landscape develops metastable minima in which the induced moments remain well-aligned while the permanent moment is misaligned (Fig. 5(c)). Relaxation therefore proceeds *via* small hops between nearby wells, as shown in Fig. 5(d) (bottom). In the low field case, the induced moments act as a magnetic anisotropy and so introduce a preferred rotation direction that depends on the roll state of the dimer. This can be observed in the low-field trajectory of Fig. 5(b). Here the dimer rotates in the plane clockwise three times, rolls around its dimer axis at *t* ≈ 30 s and then rotates in the opposite direction. Fig. 5(d) highlights the origin of this behaviour: in this case there is a strong barrier to rotate clockwise and so all hops are anticlockwise. However, by rolling the dimer π radians around the dimer axis, the dimer sits on the other side of the maxima on field flip, such that the preferred rotation direction can be reversed.

The two different regimes of behaviour at high and low field can be characterised in more detail *via* the mean squared step size upon field inversion for a trajectory, 〈(Δ*θ*)^2^〉, as shown in Fig. 6(a). This exhibits a sharp crossover from full flipping of the dimer to small hops as |*B*| is increased, with the mixed trajectory intermediate between these cases. The bifurcation in field reversal response can be captured quantitatively by a spinodal condition in the effective yaw landscape: at a critical field magnitude |*B*| = *B*_crit_, a metastable minimum on a given branch disappears. This is defined by:7
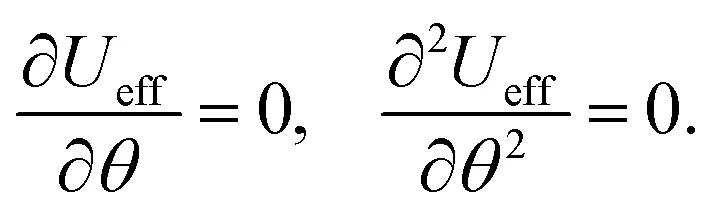


**Fig. 6:**
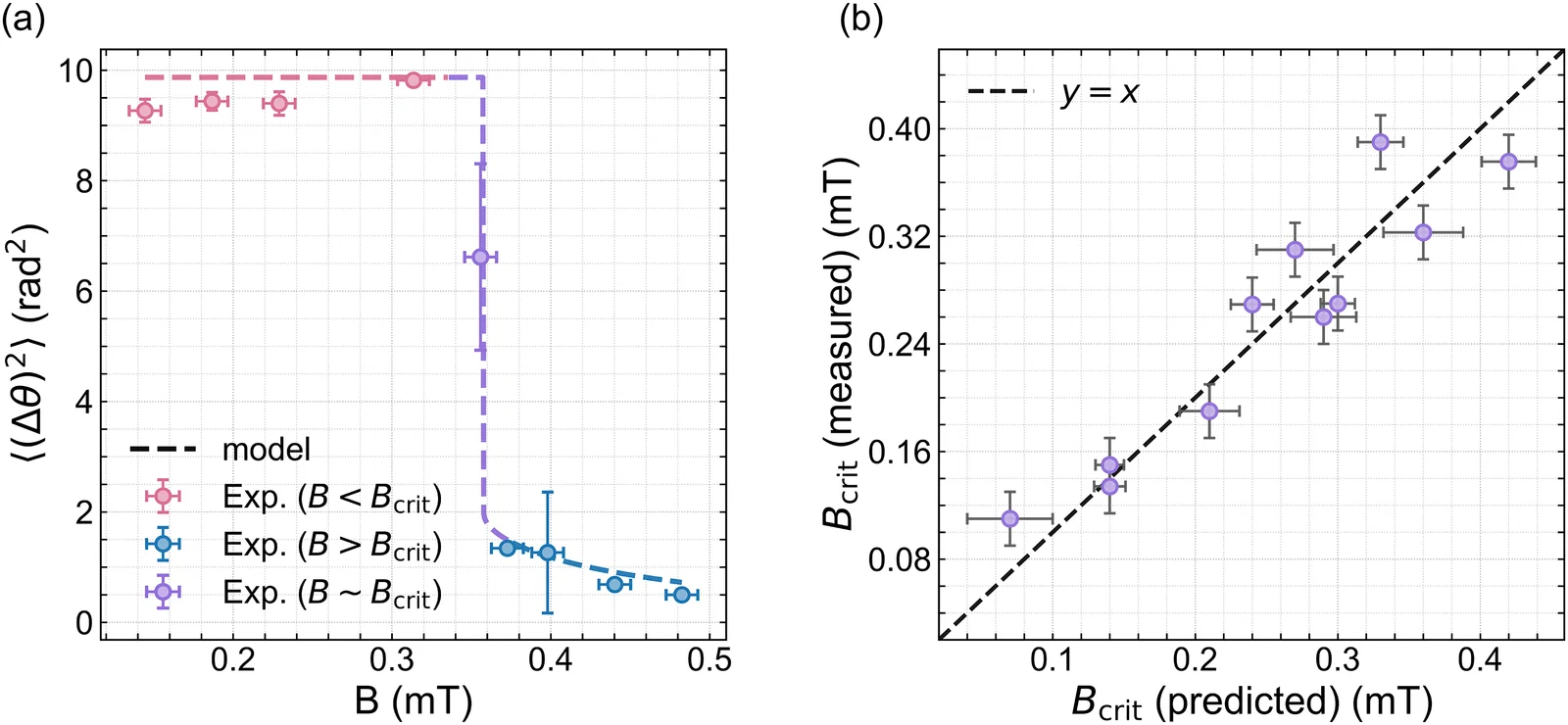
(a) Mean squared angular step size, 〈(Δ*θ*)^2^〉, as a function of *B*, demonstrating the bifurcation in switching behaviour. Error bars denote the standard error of the mean over switching events at each field (*n* ≈ 10–20 events). Predicted theoretical variation (*α* = 1.2 rad, *R* = 0.4) is shown as a dashed line. (b) Comparison of the experimentally measured *B*_crit_ and prediction from eqn (8) for multiple dimers. Error bars indicate standard errors in extracted experimental *B*_crit_ and in the predicted value obtained by propagating uncertainties in fitted parameters. The variation in *B*_crit_ can be attributed to heterogeneity in permanent moment strengths and orientations across dimers.

For eqn (5), this yields:8
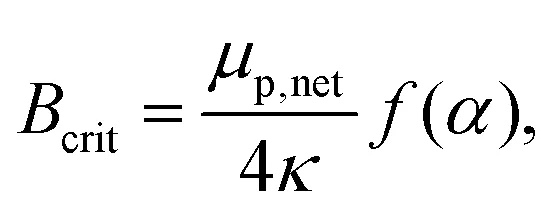
with the angular shape factor:

9 *f*(*α*) = (|cos(*α*)|^2/3^ + |sin(*α*)|^2/3^)^3/2^.

Eqn (9) has the astroid form familiar to the Stoner–Wohlfarth switching boundary,^31,33^ with the role of an easy-axis anisotropy replaced here by the induced dipole–dipole term ∝*B*^2^ (eqn (5)). Using *μ*_p,net_ and *α* obtained from the static well positions and *κ* from intra-well fluctuations, eqn (8) predicts *B*_crit_ for each dimer without additional fitting, in agreement with the crossover observed in 〈(Δ*θ*)^2^〉. This is shown as the dashed line in Fig. 6(b). Comparison between predicted and observed *B*_crit_ is good within error across dimers, with critical fields ranging from (0.1–1.0) mT (Fig. 6(b)).

#### 2.2.1 Single-dimer magnetometry

Taken together, the static misorientation *θ*_0_(*B*), high-field intra-well stiffness *κ*, and crossover field *B*_crit_ provide a practical route to estimating the net permanent dipole of an individual dimer from microscopy. First, at sufficiently large *B*, the local curvature of *U*_eff_(*θ*;*B*) is dominated by the induced term. By approximating the dynamics about a single minimum as an overdamped Ornstein–Uhlenbeck process, the short-time variance 〈Δ*θ*^2^〉 yields the induced stiffness *κ* for that dimer. Second, the measured well positions *θ*_0_(*B*) at one or more intermediate fields constrain *μ*_p,net_/*κ* and the in-plane angle *α via*eqn (6). Finally, the independently determined *B*_crit_ provides an additional constraint through eqn (8). In this way, combined static and flipping-field measurements act as a single-object rotational magnetometry protocol for anisotropic superparamagnetic dimers, without requiring separate bulk magnetometry. By applying this protocol, we observe permanent moments of (0.5–3.0) × 10^−16^ A m^2^ (consistent with literature values^32^) with characteristic misorientation angles ranging widely, from (0.3–2.2) rad for our measured dimers.

## 3 Conclusions

Our results show that weak effective permanent moments in nominally superparamagnetic microparticles can have measurable consequences for the orientational dynamics of even the simplest assembly, a rigid dimer. A minimal roll-yaw landscape comprising an even-in-*B* induced term and an odd-in-*B* permanent-dipole tilt quantitatively accounts for (i) the odd-in-*B* preferred orientation of magnetic–non-magnetic dimers, (ii) the bistable static yaw landscape of rigid magnetic–magnetic dimers, and (iii) the sharp crossover in switching behaviour under periodic field reversal. The same framework suggests a practical single-object protocol to extract the magnitude and in-plane orientation of the dimer's net permanent moment from direct imaging, albeit one that is most effective for large misorientation angles.

More broadly, these results identify rigid magnetic dimers as a minimal soft-matter system in which internal magnetic asymmetries are converted into multistable orientational dynamics and field-controlled switching pathways. This behaviour is relevant to the interpretation of torque-generating bead assays and to the design of simple magnetic microactuators, especially in weak-field regimes. More generally, this work shows that the microscopic magnetic heterogeneity of superparamagnetic microparticles leaves a clear dynamical fingerprint at the level of individual colloidal assemblies.

## Author contributions

J. R. N. T.: conceptualisation, methodology, investigation, data curation, formal analysis, visualisation, writing – original draft. F. J.: investigation, data curation, writing – review & editing. B. S.: formal analysis, visualisation, writing – review & editing. A. L. T.: conceptualisation, methodology, supervision, writing – review & editing.

## Conflicts of interest

There are no conflicts to declare.

## Supplementary Material

SM-022-D6SM00349D-s001

## Data Availability

All data needed to evaluate the conclusions in the paper are present in the paper and/or the supplementary information (SI). All other data are available from the corresponding author upon reasonable request. Supplementary information is available. See DOI: https://doi.org/10.1039/d6sm00349d.
