# Supplementary material for: Orientational bistability and field-controlled switching of a superparamagnetic dimer
Source: Soft Matter. 2026 Aug 14;22(37):6198–205. doi: 10.1039/d6sm00349d (PMC13626079; doi:10.1039/d6sm00349d)
Supplement: Supplementary file 1 [file SM-022-D6SM00349D-s001.pdf]

Supplementary Information (SI) for Soft Matter.
This journal is © The Royal Society of Chemistry 2026
Supplementary Information (SI) for Soft Matter.
This journal is © The Royal Society of Chemistry

Supplementary Information

Orientational bistability and field-controlled switching of a superparamagnetic dimer

J. R. N. Tett, F. Johnston, B. Sprinkle, A. L. Thorneywork

July 30, 2026

S1 Experimental protocol for the enrichment of dimers and aggregates

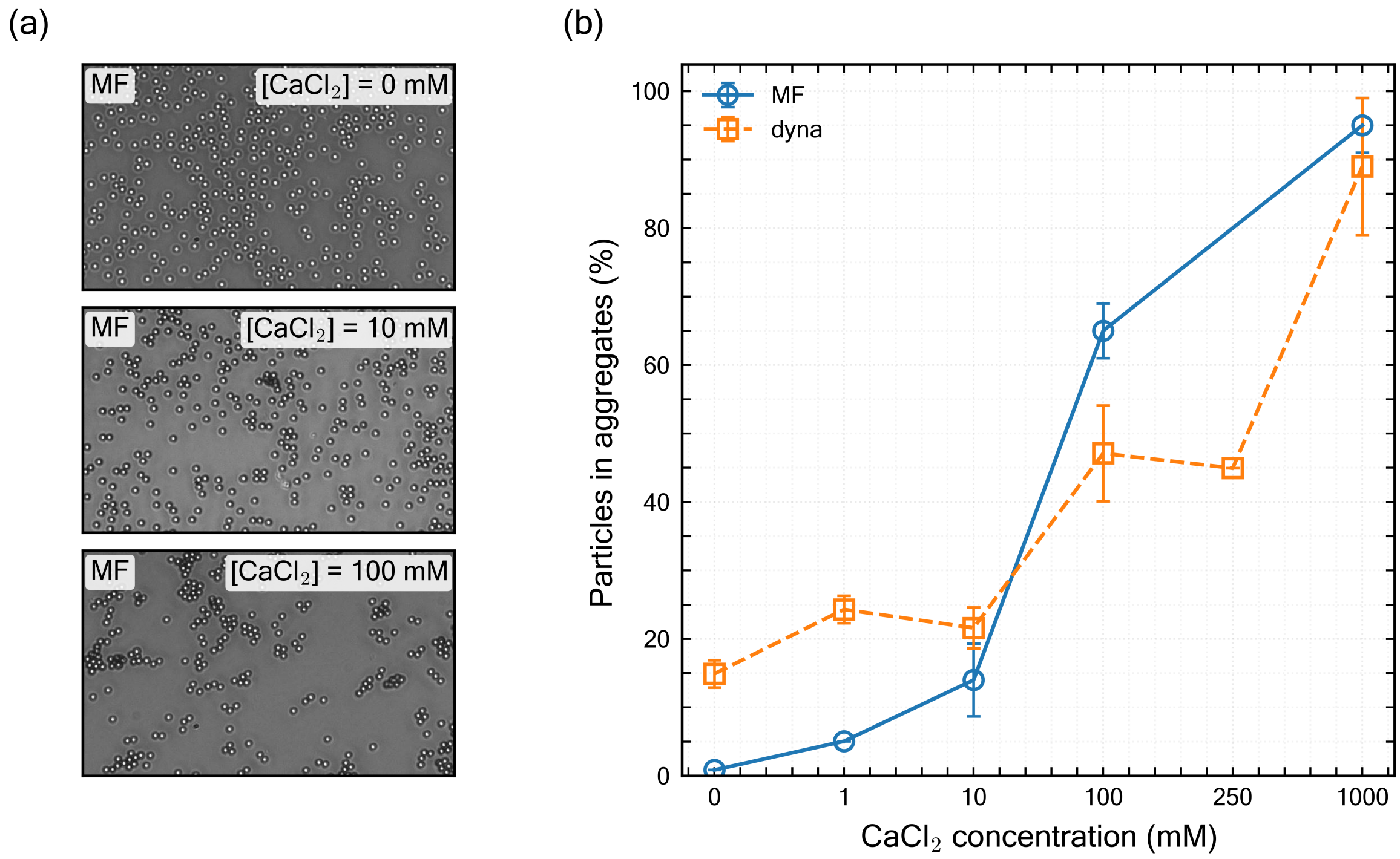

Figure S1: Enrichment of permanently bound aggregates with increasing salt concentration. (a) Representative microscopy images of melamine formaldehyde (MF) particles in aggregates as a function of $CaCl_2$ *concentration. (b) Fraction of particles in aggregates as a function of* $CaCl_2$ *concentration. Aggregates are defined as clusters containing two or more particles.*

Figure S1a shows representative microscopy images of particle suspensions prepared in different $CaCl_2$ concentrations. In the absence of $CaCl_2$, most particles remain isolated. As the concentration of $CaCl_2$ is increased, the number of permanently bound aggregates increases. This trend is observed for both magnetic particles (dynabeads) and melamine-formaldehyde particles (MF). We quantify this trend by counting the number of isolated particles and aggregates in microscopy images after washing and dilution, as plotted in Figure S1b. In all particle types, the number of aggregates (objects with ≥ 2 particles) increases with $CaCl_2$ addition for both particle types. Using this data, we determined to use a concentration of 100mM $CaCl_2$ for dimer creation.
Briefly, our protocol was as follows:

1. Particles were suspended in 100mM $CaCl_2$ and vortexed.
2. Particles were then centrifuged at 3500RCF for 20 minutes. This serves to encourage particle aggregation.

3. Particles were incubated for 12 hours and then washed three times in deionised water.
4. After the final wash, particles were resuspended to the final, low packing fractions ($\sim 0.1\%$) using deionised water and loaded into the sample cell.

This protocol does not produce dimers deterministically, but rather produces a dilute mixture containing isolated particles, dimers and larger aggregates. In experiments, we select isolated dimers and verify their rigidity directly from the tracked bead positions. The dimers considered in this paper show narrow bond-length distributions with no systematic field dependence, confirming that they remain rigid throughout measurements (Section S4).

S2 Dimer Positional Diffusion

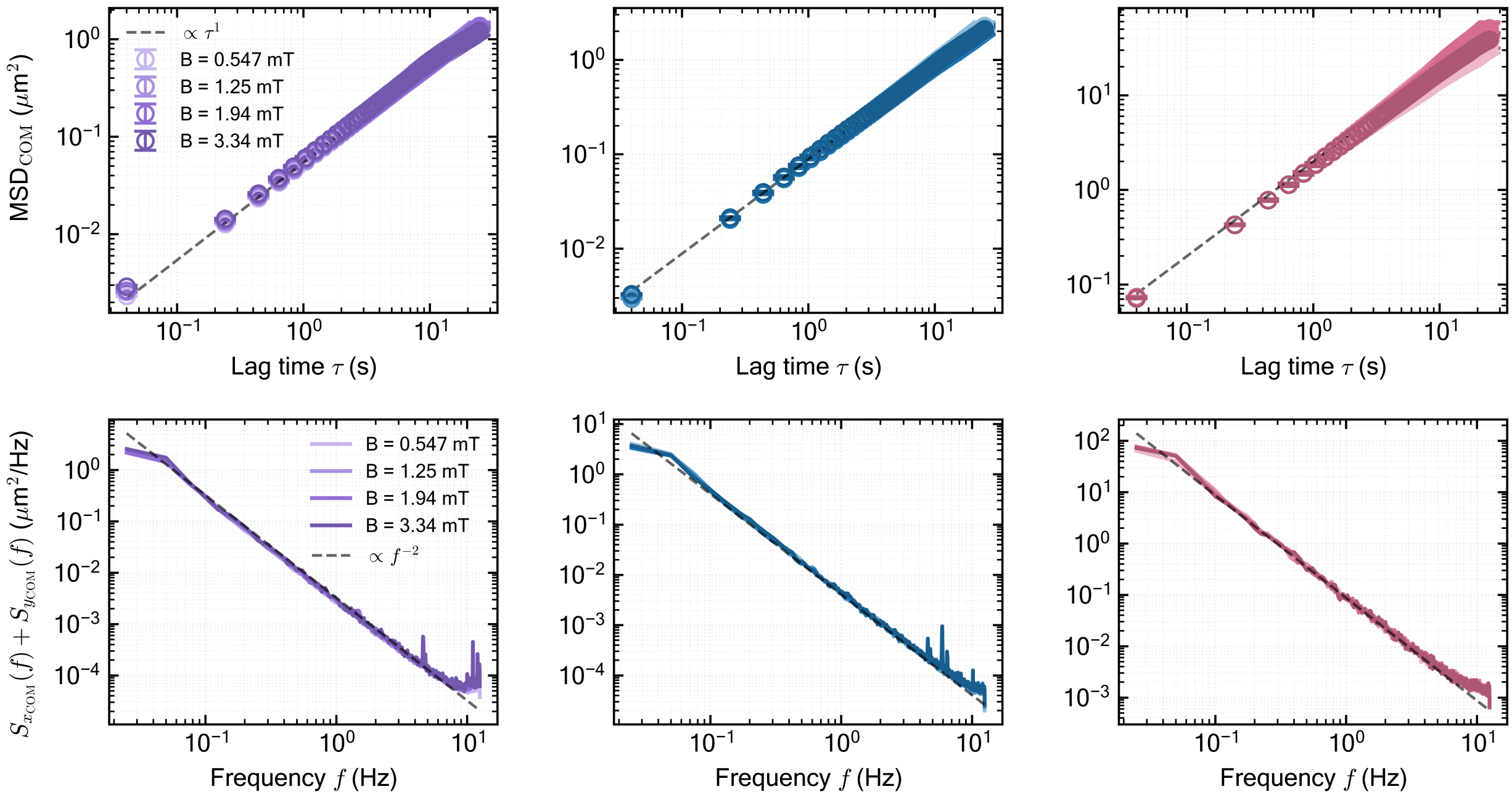

Figure S2: ***Centre-of-mass motion is purely diffusive and field-independent.*** *(Top) Mean-squared displacement (MSD) of the dimer centre-of-mass for four different fields for i) Magnetic-magnetic, reversibly (field) bonded dimers, ii) Magnetic-magnetic rigid dimers and iii) Magnetic-non-magnetic rigid dimers. (Bottom) The equivalent positional power spectral densities. In all cases, the MSDs scales as $\propto t^1$, and PSDs as $\propto 1/f^2$. No change in offset with field implies a diffusion coefficient that is invariant with B.*

Figure S2 shows that the dimer centre-of-mass (CoM) remains in the normal-diffusive regime across all applied fields and for all dimer classes studied. In the time domain, the MSD grows linearly with lag time (shown as a dashed line), consistent with $\langle \Delta r^2(t) \rangle = 4Dt$. In the frequency domain, the positional PSD follows the Brownian form, $S_r(f) \propto 1/f^2$. Importantly, increasing B does not produce any systematic change in the MSD slope or the PSD amplitude, indicating that the translational diffusion coefficient D is field invariant within experimental uncertainty. This is consistent with a spatially uniform field over the imaging region: the magnetic field introduces torques on the dimer without generating net translational forces, so no additional field-dependent drift is observed in the CoM dynamics.

S3 Dimer Orientational Diffusion

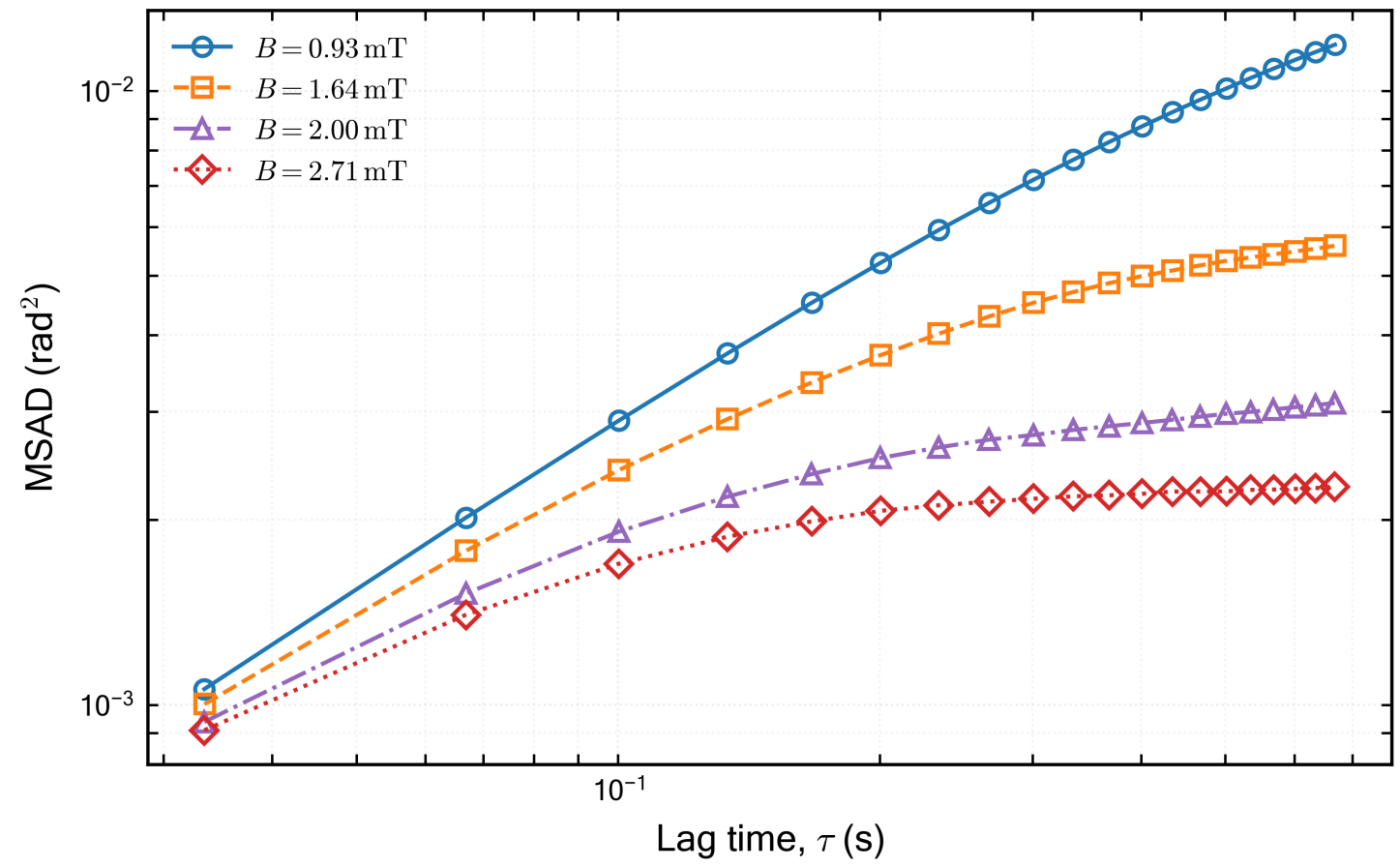

Figure S3: Typical mean-squared angular displacements (MSADs) for a rigid dimer at multiple applied magnetic fields.

Figure S3 shows the mean-squared angular displacements for a rigid dimer at different applied magnetic fields. At short lag times, all curves display the same linear scaling, $\langle \Delta\theta^2(\tau) \rangle \propto \tau$, consistent with free rotational Brownian motion before the magnetic restoring torque becomes significant. At longer lag times, the MSAD deviates from this diffusive behaviour and approaches a plateau, indicating confinement of the dimer orientation within a field-induced potential well. As the field strength increases, this crossover occurs at increasingly shorter lag times, consistent with more rapid suppression of angular excursions in a steeper orientational landscape. These data are therefore consistent with rotational Brownian motion in a confining potential whose stiffness increases with applied field.

S4 Dimer Rigidity - Bond Length

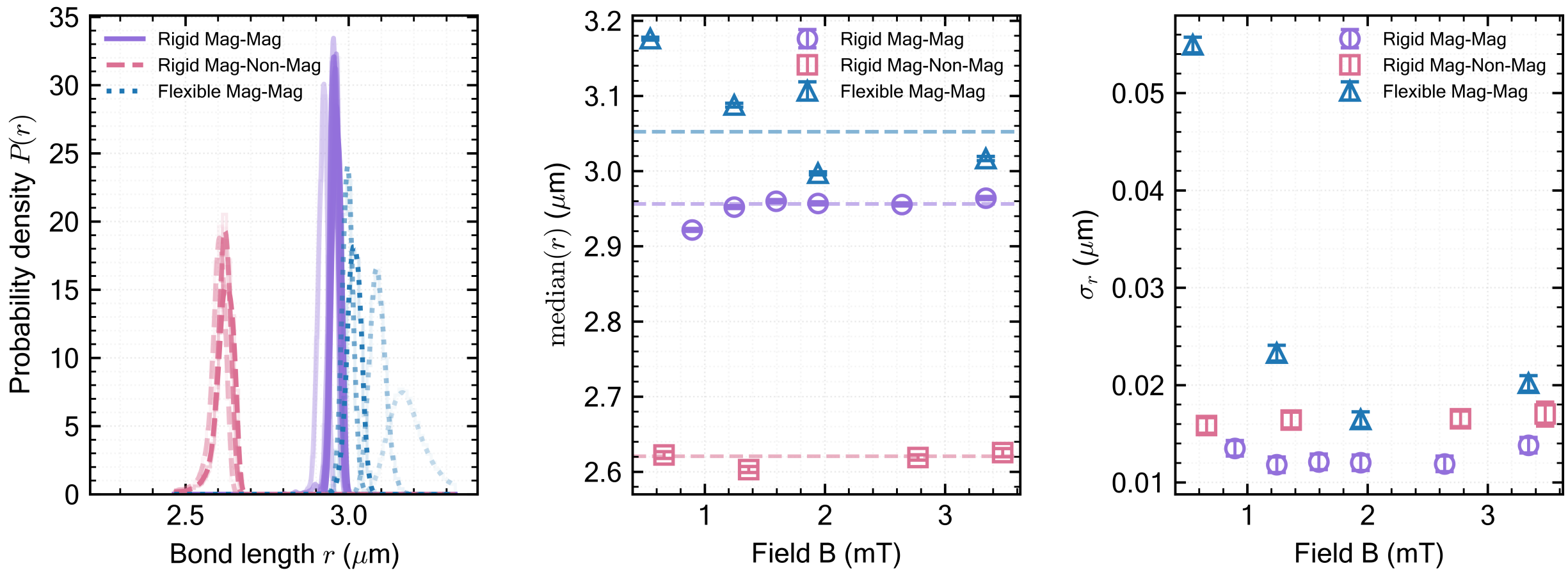

Figure S4: ***Bond-length fluctuations are minimal for rigid dimers.*** *(i) Representative bond-length distributions $P(r)$ for each dimer class at different fields. The reversibly (field) bonded magnetic-magnetic dimers have significantly larger fluctuations at low field than for either rigid construct. (ii) Median bond length as a function of applied field for flexible magnetic-magnetic (blue), rigid magnetic-magnetic (purple) and rigid magnetic-non-magnetic (pink) dimers. The median separation is essentially field-invariant for rigid dimers, while the flexible dimers show a systematic field dependence consistent with changes in bond strength. (iii) Variance of $\ell(t)$ vs. field for each dimer type, showing significantly higher bond-length variability for flexible magnetic-magnetic dimers at low fields.*

Figure S4a shows the distribution of measured bond lengths for magnetic-magnetic dimers (rigid (purple) and field-induced (blue) linkages) and for magnetic-non-magnetic dimers (rigid, pink). Rigid magnetic-magnetic

and magnetic-non-magnetic dimers exhibit nearly constant inter-bead separation: the median bond length (Figure S4b) does not vary systematically with field and the variance of $\ell(t)$ remains small across all B (Figure S4c). In contrast, reversibly (field) bonded magnetic-magnetic dimers display appreciable bond-length fluctuations and a clear field dependence in both the median and variance of $\ell(t)$ at lower fields. This is consistent with field-variable bond stiffness. This provides evidence that the rigid dimers in our study have a fixed bond throughout the experimental measurements.

S5 Mutual Induction and Cross Terms

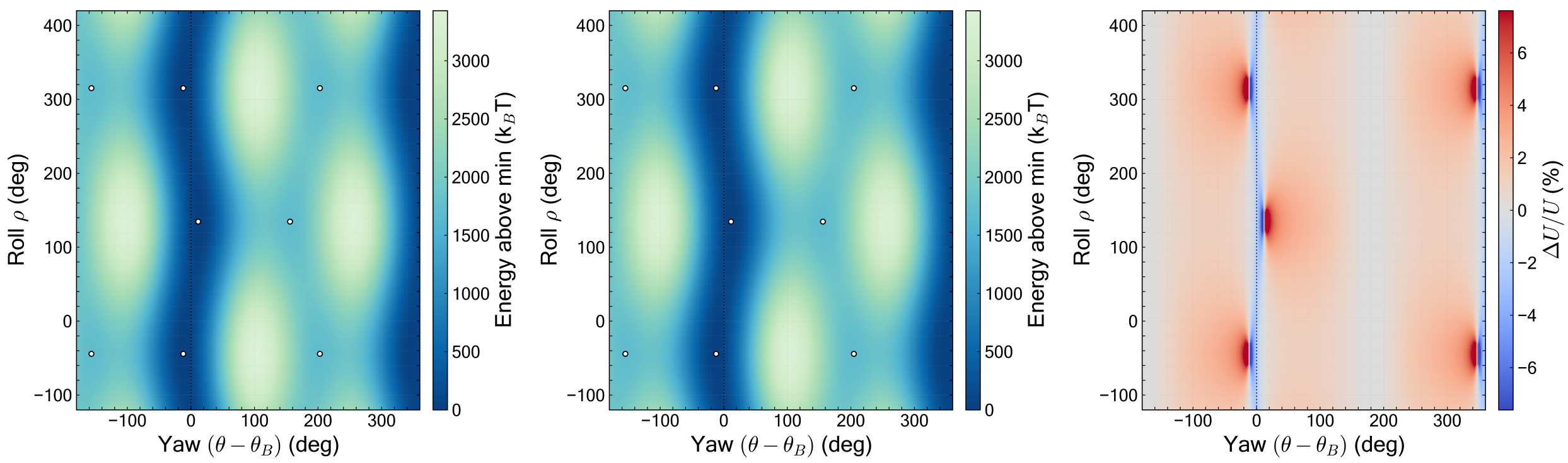

Figure S5: ***Mutual induction is a weak correction for*** $U(\theta, \rho; B)$***.*** *Left: The full roll-yaw energy landscape* $U(\theta, \rho; B)$ *for a rigid dimer with permanent moments* $\left[|\boldsymbol{\mu}_{p,net}|, arg(\boldsymbol{\mu}_{p,net})\right] = \left[6.6 \times 10^{-16} Am^2, 45°\right]$*. The landscape is computed with self-consistent updates of the induced dipole moments in the local field (defined by the external field and the permanent and induced dipoles from the other bead). Middle: The same roll-yaw energy landscape computed with only induced-induced and permanent-field interactions. Induced moments are evaluated from the external field only. Right: Local percentage difference residuals, with a small floor to prevent divergence when* U *is close to zero.*

Figure S5 compares the full energy landscape computed with self-consistent dipoles (Figure S5a) to a simplified calculation in which the induced moments are evaluated once from the external field (i.e. neglecting mutual-induction feedback - Figure S5b). The two landscapes are visually indistinguishable at the level relevant for our analysis: the locations of minima, barrier structure and overall angular dependence are unchanged, and the pointwise energy differences are typically only a few percent (Figure S5c). Apparent "spikes" in the percent-difference map occur only in regions where the absolute energy is close to zero, so a small absolute mismatch is amplified by normalisation rather than indicating a physically meaningful discrepancy. With this in mind, we treat mutual induction and the associated induced-permanent cross terms as a weak correction and use the simplified induced dipole-permanent dipole picture throughout (Section S8).

S6 Single particle response to field inversion

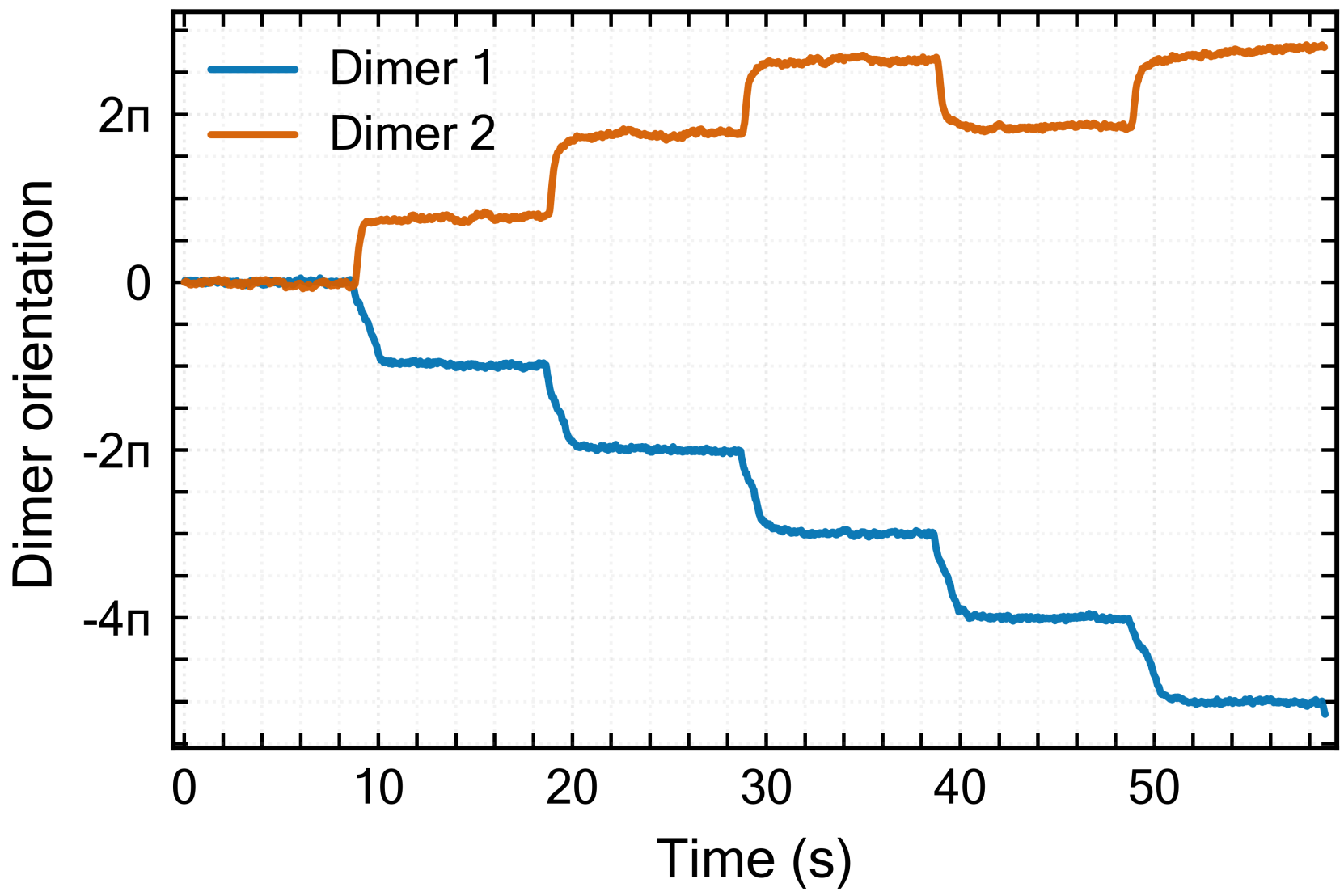

Figure S6: ***Magnetic microparticles are odd-in-field.*** *Representative orientational time series data of two rigid magnetic-non-magnetic dimers under repeated reversals of the applied field. Each field inversion produces a* $\sim \pi$ *reorientation of the dimer body, consistent with a body-fixed magnetic moment. A purely induced response is even in* B *and so could not, by itself, generate such a relaxation response. Notably, different dimers show different responses to field inversion with respect to direction of rotation.*

Figure S6 shows that rigid magnetic-non-magnetic dimers undergo repeatable, step-like reorientations of $\sim \pi$ radians when the external field is reversed. This behaviour is consistent with a magnetic contribution that is fixed to the dimer body (a permanent moment). A purely induced dipole response is even in B and so, on its own, could not account for the field-inversion response. This serves as direct evidence for an odd-in-B (permanent moment) term in the single-particle orientational potential and is consistent with the orientational bistability and low-field inversion responses in rigid magnetic-magnetic dimers. Interestingly, magnetic-non-magnetic dimers can show a preferred direction of rotation under repeated field reversals. The type and extent of this preference varies between dimers, as illustrated by the comparison of dimers 1 and 2. This dimer-to-dimer variability suggests additional bead-specific asymmetry beyond the minimal fixed-dipole picture, potentially arising from a further internal magnetic anisotropy of individual nanoparticles. A detailed analysis of this directional preference will be addressed separately.

S7 Interaction Stiffness variation with field

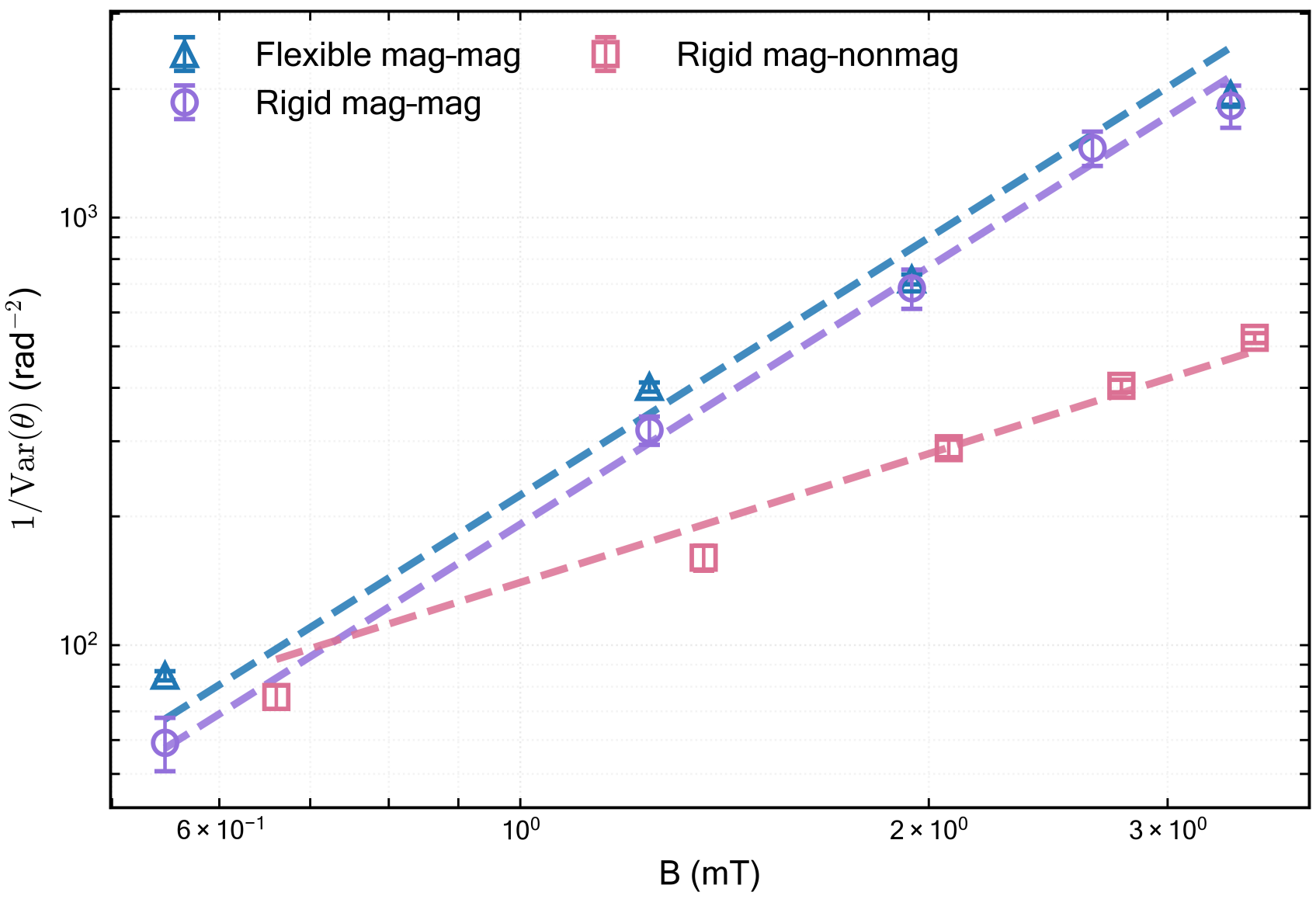

Figure S7: ***Interaction stiffness scalings from intra-well orientational fluctuations for different dimer types.*** *Experimental data are shown as points, while dashed lines show fits to the data. These scale as $\propto B^2$ for magnetic-magnetic dimers and $\propto B^1$ for magnetic-non-magnetic dimers.*

A robust estimate of the local interaction stiffness can be obtained directly from $\theta(t)$. Small fluctuations within an orientational well are well-approximated as overdamped harmonic motion:

$$U_{\text{eff}}(\theta) \simeq U_0 + \frac{1}{2}\kappa_{\text{loc}}\,(\theta - \theta_{\text{min}})^2\,, \tag{S1}$$

so that local equilibrium implies a stationary variance

$$\mathrm{Var}(\theta) \equiv \left\langle (\theta - \langle\theta\rangle)^2 \right\rangle \simeq \frac{k_B T}{\kappa_{\text{loc}}}. \tag{S2}$$

We therefore define a variance-based stiffness estimator

$$\kappa_{\text{var}} \equiv \frac{k_B T}{\mathrm{Var}(\theta)}, \qquad \text{so that} \qquad \frac{1}{\mathrm{Var}(\theta)} = \frac{\kappa_{\text{var}}}{k_B T}. \tag{S3}$$

Stationary-segment protocol. To ensure that Eq. (S2) probes intra-well fluctuations rather than rare switching events or slow drift of the mean angle, we compute $\mathrm{Var}(\theta)$ only on stationary regions of $\theta(t)$: contiguous time intervals in which the trajectory remains in a single well and the local mean angle is approximately constant. For each trajectory we segment $\theta(t)$ into windows and retain those windows that (i) do not contain a switching event between wells (where relevant) and (ii) pass a simple stationarity check (small change in window mean relative to the intra-window standard deviation). For rigid magnetic-magnetic dimers, which exhibit two wells, we compute $\mathrm{Var}(\theta)$ separately within each well (i.e. conditioning on the branch) and then combine the resulting stiffness estimates. Uncertainties are obtained by bootstrap resampling of stationary windows.

Expected field scalings. Figure S7 shows the interaction stiffness for different dimer types. For magnetic-magnetic dimers whose orientational confinement is dominated by induced dipole-dipole interactions, the effective yaw potential contains an even-in-B term $U_{\text{ind}}(\theta) \propto -B^2 \cos 2\theta$, so the local curvature (and thus $1/\mathrm{Var}(\theta)$) scales as B^2. By contrast, for magnetic-non-magnetic dimers, whose orientational confinement is dominated by the interaction of a body-fixed moment with the external field, the leading term is $U_{\text{p}}(\theta) \propto -B\cos(\theta - \alpha)$, giving a local curvature that scales linearly with B.

S8 Rigid dimer interaction potential

Here, we derive the effective orientational potential used to describe rigid magnetic-magnetic dimers. We first show that two distinct in-plane permanent moments, one in each bead, are not sufficient to produce a bimodal yaw distribution owing to the linearity of the dipole-field interaction. We then introduce the roll-yaw geometry of a sedimented rigid dimer, in which a three-dimensional body-fixed moment can have a roll-dependent projection into the sedimentation plane. This additional degree of freedom provides a route to the paired yaw minima observed experimentally.

S8.1 Two permanent moments reduce to one effective moment

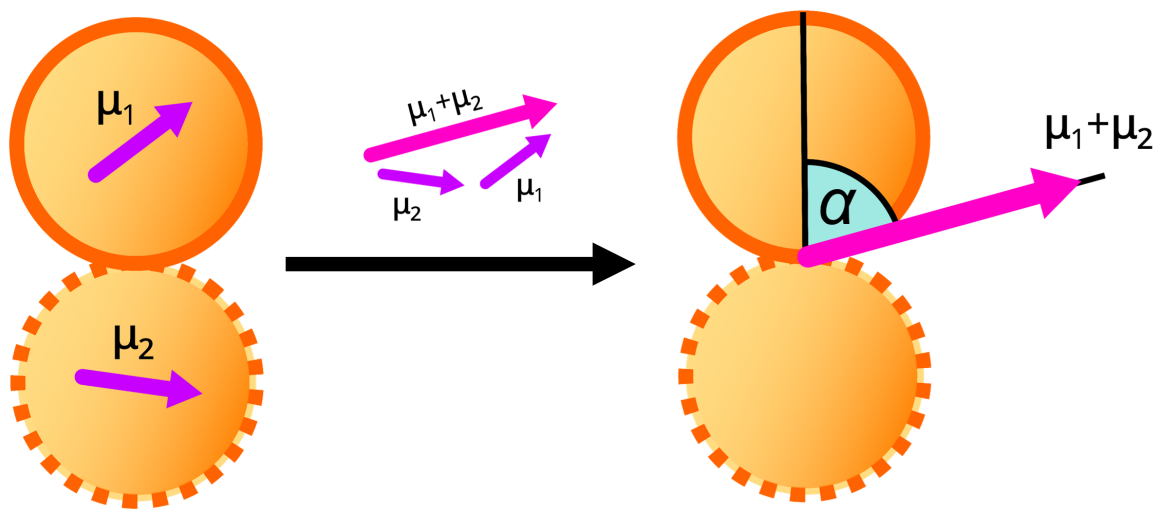

Figure S8: Each bead has a permanent moment, $\boldsymbol{\mu}_{p,1}$ and $\boldsymbol{\mu}_{p,2}$. These moments are fixed in the rigid dimer body frame and can be combined to form an effective, dimer-level permanent moment. This permanent moment can always be brought into the sedimentation plane via a roll of the dimer.

Whilst the observed bistability of rigid dimers could be hypothesised to be due to the alignment of two distinct body-fixed permanent moments, where each peak represents the alignment of one moment, this is in fact not the case. Importantly, the dipole-field energy can always be expressed in terms of a single effective in-plane permanent moment.

Let the two bead-level permanent moments be $\boldsymbol{\mu}_{p,1}$ and $\boldsymbol{\mu}_{p,2}$, both fixed in the dimer body frame. These moments are 3D vectors that could, in principle, be oriented in any way with respect to one another. Their total coupling to the applied field can be written as:

$$U_p = -\boldsymbol{\mu}_{p,1} \cdot \mathbf{B} - \boldsymbol{\mu}_{p,2} \cdot \mathbf{B} \tag{S4}$$

where $\mathbf{B}$ is the applied field. This field coupling is linear in the magnetic moment and so can be rewritten:

$$U_p = -\left(\boldsymbol{\mu}_{p,1} + \boldsymbol{\mu}_{p,2}\right) \cdot \mathbf{B} \tag{S5}$$

$$= -\boldsymbol{\mu}_{p,\text{eff}} \cdot \mathbf{B} \tag{S6}$$

Where $\boldsymbol{\mu}_{p,\text{eff}} = \boldsymbol{\mu}_{p,1} + \boldsymbol{\mu}_{p,2}$, i.e. an effective permanent moment obtained by the vector sum of the two, 3D moments. This effective moment makes an angle α to the dimer axis (as seen in Figure S8). As the dimer rolls, $\boldsymbol{\mu}_{p,\text{eff}}$ traces out a cone with half-angle α. With $\boldsymbol{\mu}_{p,\text{eff}}$ in-plane, the permanent-field coupling then becomes:

$$U_p(\theta) = -\mu_{p,\text{eff}} B \cos(\theta - \alpha) \tag{S7}$$

This potential has a single preferred yaw orientation over a 2π interval so, whilst two distinct in-plane, bead-level permanent moments can shift the preferred yaw angle, through the direction of $\boldsymbol{\mu}_{p,\text{eff}}$, they cannot generate a bimodal yaw distribution. Rationalising the observed bimodality therefore requires an additional rotational degree of freedom. In the model below, this is supplied by roll around the dimer axis.

S8.2 Geometry and magnetic moments

We model a rigid sedimented dimer with two relevant rotational degrees of freedom: (i) the in-plane yaw angle θ of the bond vector relative to the laboratory $\hat{\mathbf{y}}$ axis, which is the field direction, and (ii) a roll angle ρ about the dimer axis. Pitch is strongly suppressed by gravity/wall confinement and is neglected.
Let the unit bond vector in the sample plane be:

$$\hat{\mathbf{n}} = \sin\theta\ \hat{\mathbf{x}} + \cos\theta\ \hat{\mathbf{y}} \tag{S8}$$

So that $\theta = 0$ corresponds to the dimer axis aligned with the applied field. We define the orthonormal triad $\hat{\mathbf{n}}, \hat{\mathbf{t}}, \mathbf{z}$ with:

$$\hat{\mathbf{t}}(\theta) = \hat{\mathbf{z}} \times \mathbf{n}(\theta) = -\cos(\theta)\ \hat{\mathbf{x}} + \sin(\theta)\ \hat{\mathbf{y}} \tag{S9}$$

A roll rotation by ρ rotates vectors in the plane perpendicular to $\hat{\mathbf{n}}$. A convenient parameterisation of the net permanent moment in the laboratory frame is therefore:

$$\boldsymbol{\mu}_{p,\mathrm{net}}(\theta, \rho) = \mu_{\|}\ \hat{\mathbf{n}}(\theta) + \mu_{\perp}\ \hat{\mathbf{e}}_{\perp})(\theta, \rho) \tag{S10}$$

$$= \cos(\rho)\ \hat{\mathbf{t}}(\theta) + \sin(\rho)\ \hat{\mathbf{z}} \tag{S11}$$

where $\mu_{\|}$ and $\mu_{\perp}$ are the components parallel and transverse to the dimer axis. We take the applied field to be uniform and in-plane,

$$\mathbf{B} = B\ \hat{\mathbf{y}} \tag{S12}$$

and assume each bead has a dominant induced moment aligned with the field,

$$\boldsymbol{\mu}_{\mathrm{ind},i} = \chi B \hat{\mathbf{y}}, \qquad i = 1, 2 \tag{S13}$$

In the isotropic, linear-response regime.

S8.3 2D roll-yaw energy

Retaining only orientation-dependent terms, the total energy is written as a sum of a permanent-field term and an induced dipole-dipole term:

$$U(\theta, \rho) = -\boldsymbol{\mu}_{p,\mathrm{net}}(\theta, \rho) \cdot \mathbf{B} + \boldsymbol{\mu}_{\mathrm{ind},2} \tag{S14}$$

where $\mathbf{T}_{12} = \frac{\mu_0}{4\pi r^3}(\mathbf{I} - 3\hat{\mathbf{r}}\hat{\mathbf{r}})$ is the dipolar coupling tensor at fixed bead separation $r \simeq \sigma$. The tensor depends on the bead-centre separation vector and therefore on θ, but not on the roll angle ρ.

S8.4 Eliminating roll: effective 1D yaw potential

Experimentally we measure yaw statistics only, so we eliminate ρ to obtain an effective 1D potential for θ. A general elimination is the marginal (free-energy) potential

$$U_{\mathrm{eff}}(\theta) \equiv -k_B T \ln\left(\int_0^{2\pi} d\rho\, e^{-\beta U(\theta, \rho)}\right), \qquad \beta = (k_B T)^{-1}. \tag{S15}$$

In the regime where roll is fast and sharply localised within one branch (or equivalently when $U(\theta, \rho)$ is steep in ρ), this reduces to a branch-wise minimisation (adiabatic elimination),

$$U_{\mathrm{eff}}^{(\mathrm{branch})}(\theta) \simeq U(\theta, \rho_{\min}(\theta)), \qquad \rho_{\min}(\theta) = \arg\min_{\rho} U(\theta, \rho). \tag{S16}$$

Because the sedimentation geometry "cuts" an otherwise continuous precession manifold, there are two symmetry-related roll branches; following one branch yields a single-well $U_{\mathrm{eff}}(\theta)$, and applying the glide symmetry generates the partner well on the other branch.

S8.5 Permanent-field contribution

Using $\hat{\mathbf{n}} \cdot \hat{\mathbf{y}} = \sin\theta$, $\hat{\mathbf{t}} \cdot \hat{\mathbf{y}} = \cos\theta$, and $\hat{\mathbf{z}} \cdot \hat{\mathbf{y}} = 0$, we obtain:

$$\boldsymbol{\mu}_{p,\mathrm{net}}(\theta, \rho) \cdot \mathbf{B} = B\Big[\mu_{\|}(\hat{\mathbf{n}} \cdot \hat{\mathbf{y}}) + \mu_{\perp}(\hat{\mathbf{e}}_{\perp} \cdot \hat{\mathbf{y}})\Big] \tag{S17}$$

$$= B\Big[\mu_{\|} \sin\theta + \mu_{\perp} \cos\rho\, \cos\theta\Big]. \tag{S18}$$

On a given roll branch, $\cos\rho$ is approximately constant (e.g. $\cos\rho = \pm 1$ for branches related by $\rho \to \rho + \pi$), so the combination

$$\mu_{\|} \sin\theta + \mu_{\perp} \cos\rho\, \cos\theta \tag{S19}$$

can be written as a single phase-shifted cosine:

$$\mu_{\parallel} \sin\theta + \mu_{\perp} \cos\rho \, \cos\theta = \mu_{p,\mathrm{net}} \cos(\theta - \alpha), \tag{S20}$$

where the effective magnitude and in-plane misorientation are defined by matching coefficients:

$$\mu_{p,\mathrm{net}} = \sqrt{\mu_{\parallel}^2 + (\mu_{\perp} \cos\rho)^2}, \tag{S21}$$

$$\cos\alpha = \frac{\mu_{\perp} \cos\rho}{\mu_{p,\mathrm{net}}}, \qquad \sin\alpha = \frac{\mu_{\parallel}}{\mu_{p,\mathrm{net}}}. \tag{S22}$$

Thus the permanent-field energy on a fixed roll branch is

$$U_p(\theta) \equiv -\boldsymbol{\mu}_{p,\mathrm{net}} \cdot \mathbf{B} = -\mu_{p,\mathrm{net}} \, B \, \cos(\theta - \alpha). \tag{S23}$$

S8.6 Induced dipole-dipole contribution becomes $\cos 2\theta$

For two induced moments aligned with the field, $\boldsymbol{\mu}_{\mathrm{ind},1} = \boldsymbol{\mu}_{\mathrm{ind},2} = \chi B \, \hat{\mathbf{y}}$, the dipole-dipole energy can be written (up to an additive constant) as:

$$U_{\mathrm{ind}}(\theta) \propto -(\chi B)^2 \left[3(\hat{\mathbf{y}} \cdot \hat{\mathbf{r}})^2 - 1\right], \tag{S24}$$

where $\hat{\mathbf{r}}$ is the unit separation vector between bead centres. For a rigid dimer, $\hat{\mathbf{r}}$ is along the bond direction, $\hat{\mathbf{r}} = \hat{\mathbf{n}}(\theta)$, so $\hat{\mathbf{y}} \cdot \hat{\mathbf{r}} = \sin\theta$ and

$$3(\hat{\mathbf{y}} \cdot \hat{\mathbf{r}})^2 - 1 = 3\sin^2\theta - 1 = \frac{3}{2}(1 - \cos 2\theta) - 1 = \mathrm{const.} - \frac{3}{2}\cos 2\theta. \tag{S25}$$

Dropping constants, we therefore obtain the leading yaw dependence

$$U_{\mathrm{ind}}(\theta) = -\kappa B^2 \cos(2\theta), \tag{S26}$$

where $\kappa = \frac{3\mu_0 \chi^2}{8\pi r^3}$, the interaction stiffness of the induced-induced dipole interaction.

S8.7 Final effective 1D yaw potential

Combining Eqs. (S23) and (S26), the effective yaw potential on a single roll branch is

$$U_{\mathrm{eff}}(\theta; B) = -\kappa B^2 \cos(2\theta) \; - \; \mu_{p,\mathrm{net}} \, B \cos(\theta - \alpha) \tag{S27}$$

This has the same mathematical structure as a Stoner-Wohlfarth energy: the induced interaction provides an even-in-B twofold anisotropy ($\propto B^2 \cos 2\theta$) and the permanent moment provides an odd-in-B Zeeman-like tilt ($\propto B \cos(\theta - \alpha)$). The second roll branch is generated by the glide symmetry $\rho \to \rho + \pi$ (which flips $\cos\rho$ and hence shifts the effective phase), producing the partner minimum and yielding the experimentally observed bimodal $P(\theta)$.
